# Developing a Health Equity Impact Assessment ‘Indigenous Lens Tool’ to address challenges in providing equitable cancer screening for indigenous peoples

**DOI:** 10.1186/s12889-023-16919-7

**Published:** 2023-11-15

**Authors:** Naana Afua Jumah, Alethea Kewayosh, Bernice Downey, Laura Campbell Senese, Jill Tinmouth

**Affiliations:** 1 Northern Ontario School of Medicine University, Thunder Bay, ON Canada; 2 University of Toronto, Toronto, ON Canada; 3 Ontario Health, Toronto, ON Canada; 4 McMaster University, Hamilton, ON Canada; 5 Sunnybrook Health Sciences Centre, Toronto, ON Canada

**Keywords:** Cancer screening, Health equity, Indigenous health, Human rights, Health Equity Impact Assessment (HEIA) Tool, Social determinants of health

## Abstract

**Background:**

In spite of past efforts to increase screening uptake, the rates of screening-detectable cancers including breast, cervical, colorectal and lung are rising among Indigenous persons in Ontario compared to other Ontarians. The Ontario Ministry of Health has an equity framework, the Health Equity Impact Assessment (HEIA) Tool, that was developed to guide organizations in the provision of more equitable health and social services. Although the HEIA Tool identifies that the health of Indigenous persons may benefit from more equitable provision of health and social services, it provides very little specific guidance on how to apply the HEIA Tool in a culturally relevant way to policies and programs that may impact Indigenous peoples.

**Discussion:**

Guided by the Calls to Action from the Truth and Reconciliation Commission of Canada and the United Nations Declaration on the Rights of Indigenous Peoples, an Indigenous Lens Tool was developed through a collaborative and iterative process with stakeholders at Cancer Care Ontario and with representatives from Indigenous community-based organizations. The Indigenous Lens Tool consists of four scenarios, with supporting documentation that provide context for each step of the HEIA Tool and thereby facilitate application of the equity framework to programs and policies. The document is in no way meant to be comprehensive or representative of the diverse health care experiences of Indigenous peoples living in Canada nor the social determinants that surround health and well-being of Indigenous peoples living in Canada. Rather, this document provides a first step to support development of policies and programs that recognize and uphold the rights to health and well-being of Indigenous peoples living in Canada.

**Conclusions:**

The Indigenous Lens Tool was created to facilitate implementation of an existing health equity framework within Cancer Care Ontario (now Ontario Health). Even though the Indigenous Lens Tool was created for this purpose, the principles contained within it are translatable to other health and social service policy applications.

## Background

While historically uncommon in Indigenous populations in Ontario, Canada, cancer is now a significant cause of morbidity and mortality. For many ‘screening-detectable’ cancers (i.e., breast, cervical, colorectal and lung) in Ontario, the incidence appears either to be rising or above that of other Ontarians and the survival rate appears to be worse for First Nations, Inuit and Métis (FNIM) persons compared to other Ontarians [1–6].

Routine cancer screening for these four cancers can improve cancer health outcomes [7–14], including cancer-related death. In Ontario, there are organized cancer screening programs for breast, cervix and colorectal cancer and in 2021, an organized lung cancer screening program in high-risk persons was launched. Organized cancer screening aims to deliver high quality screening to the entire target population in a coordinated fashion that is integrated into the broader health care system [15]. At the start of the work described in this paper, Cancer Care Ontario (CCO), an agency of the provincial government of Ontario, was responsible for overseeing the province’s organized cancer screening programs. In 2019, CCO was incorporated into Ontario Health. Ontario Health is an umbrella agency intended to administer and integrate key components of Ontario’s health care system.

In 2014, a research program was undertaken with the aim of increasing cancer screening participation in Indigenous communities in Ontario by understanding the health system, community and individual factors that impact access to and uptake of cancer screening. The research project was comprised of three aims: (1) An analysis of policies relevant to cancer screening in Indigenous peoples in Ontario [16]; (2) An analysis of the outcomes of previous projects within CCO to improve screening in under-screened and never-screened individuals; and (3) Community engagement to understand local factors that impact uptake of cancer screening [17, 18]. An overarching theme that arose from this research program was the lack of application of an Indigenous lens and the lack of an equity focus in most aspects of the cancer screening system.

The Ontario Ministry of Health has an existing equity framework, the Health Equity Impact Assessment (HEIA) Tool, that was developed to guide organizations in the provision of more equitable health and social services [19]. Although the HEIA Tool identifies Indigenous peoples living in Ontario as a population that may benefit from more equitable provision of health and social services, it provides very little specific guidance on how to apply the HEIA Tool in a culturally relevant way to policies and programs that may impact Indigenous peoples. Furthermore, the HEIA Tool treats Indigenous peoples as a uniform group rather than recognizing that there is diversity within First Nations, Inuit and Métis communities. As a result, the research team developed an Indigenous Lens Tool supplement to the HEIA Tool to address the gaps in the existing guidance.

### Main text

#### Indigenous ways of knowing

Prior to colonization, Indigenous peoples in Canada had established knowledge systems, cultural practices, values and beliefs. This included a rich diversity of knowledges and practices that promoted well-being. Colonization interrupted and de-valued these traditional ways of knowing [20, 21]. The Truth and Reconciliation Commission (TRC) Call to Action 18 challenges us, through public institutions, to affirm Indigenous peoples’ rights to health care including health care based on traditional belief systems [22]. TRC Principle 5 challenges us to “create a more equitable and inclusive society by closing gaps in social, health, and economic outcomes that exist between Aboriginal and non-Aboriginal Canadians”. TRC Principle 7 affirms the value of “the perspectives and understandings of Aboriginal Elders and Traditional Knowledge Keepers of the ethics, concepts and practices of reconciliation” [23].

In keeping with the spirit of reconciliation, health equity models and assessment tools targeted for use with Indigenous peoples must be inclusive of Indigenous knowledges and values regarding health and well-being. For example, wholism, reciprocity, resiliency, inter-dependency, respect, collectivity and relationality are values that are represented in many distinct Indigenous worldviews and may not be well reflected in or compatible with a person-centered, individualistic approach to health equity. Several documents, grounded in Indigenous worldviews, provided the foundational framework for the development of the HEIA Indigenous Lens Tool (Table 1).

**Table 1:** Guiding documents

**Ontario Ministry of Health**
• Health Equity Impact Assessment (HEIA) Tool Template https://www.health.gov.on.ca/en/pro/programs/heia/docs/template.pdf • Health Equity Impact Assessment Workbook https://www.health.gov.on.ca/en/pro/programs/heia/docs/workbook.pdf
**Ontario Health (Cancer Care Ontario)**
• First Nations, Inuit, Métis and Urban Indigenous Cancer Strategy (https://www.cancercareontario.ca/en/cancer-care-ontario/programs/aboriginal-programs/indigenous-cancer-strategy) • Ontario Cancer Plan V https://www.cancercareontario.ca/en/cancerplan
**First Nations Information Governance Committee**
• OCAP^®^ Principles (Ownership, Control, Access, Possession) https://fnigc.ca/sites/default/files/docs/ocap_path_to_fn_information_governance_en_final.pdf
**Inuit Tapiriit Kanatami and Nunavut Research Institute**
• Negotiating Research Relationships with Inuit Communities, A Guide for Researchers https://www.itk.ca/negotiating-research-relationships-guide/ • National Inuit Strategy on Research https://www.itk.ca/wp-content/uploads/2018/03/National-Inuit-Strategy-on-Research.pdf
**Métis Centre at the Native American Health Organization**
• Principles of Ethical Métis Research https://ruor.uottawa.ca/bitstream/10393/30555/1/2011_04_ethics.pdf
**Canadian Institutes of Health Research, Natural Sciences and Engineering Research Council of Canada, and Social Sciences and Humanities Research Council**
• Tri-Council Policy Statement 2 https://ethics.gc.ca/eng/documents/tcps2-2018-en-interactive-final.pdf
**Government of Canada**
• Truth and Reconciliation Commission of Canada Final Report http://www.trc.ca/about-us/trc-findings.html • Truth and Reconciliation Commission of Canada What We Have Learned: Principles of Truth and Reconciliation http://www.trc.ca/assets/pdf/Principles%20of%20Truth%20and%20Reconciliation.pdf
**United Nations**
• United Nations Declaration on the Rights of Indigenous Peoples https://www.un.org/esa/socdev/unpfii/documents/DRIPS_en.pdf

Often the terms’ Aboriginal’ and ‘Indigenous’ are used interchangeably in the ‘grey’ literature. The term ‘Indigenous’ is often preferred by academics as well as many First Nations, Inuit and Métis people in the current environment as it is aligned with a relationship to the land and legal rights as identified in the United Nations Declaration on the Rights of Indigenous Peoples (UNDRIP). However, some situate their identity more specifically by their tribal affiliation. For example, Ojibwe or Cree from a specific treaty area or land base. However, in Canada, the term Aboriginal is enshrined in the constitution. As a result, the term Aboriginal has been used when referring to legal documents or when it has been used in a source document.

#### Organizational awareness, allyship and decolonization

A key to understanding the impact of past efforts to increase screening, was the need to understand the cultural context in which those programs and policies were created. Organizations have cultures, which exist within, and are shaped by, the broader dominant culture. Organizational culture can be understood as the visible artifacts (e.g., architecture, language used, dress codes), shared values (e.g., organizational goals, strategies), and basic assumptions (e.g., taken for granted beliefs, perceptions) of an organization [24]. When an organization creates policies or provides services, the organization “often unconsciously exclude[s] others who don’t share the cultural frame of mind from which we operate [25].” Acknowledging that the culture of an individual and the culture of an organization affect decision-making is the first step to developing more equitable programs and policies. The process whereby one reflects on their relationship with Indigenous peoples living in Canada is the first step towards Indigenous allyship. The critical self-reflection that is a key component of allyship is also a step on the pathway to developing cultural humility – a commitment to reflecting on one’s own culture while learning about Indigenous cultures. Through allyship and cultural humility, one is able to move towards decolonization, support Indigenization and achieve meaningful reconciliation (Table 2) [26–28]. This journey to allyship and cultural humility varies for each of the authors as we include Indigenous women (AK and BD), settler women of European descent (LS and JT) and an immigrant woman of mixed African and European descent (NJ).

**Table 2:** Definitions from allyship to reconciliation

Principle	Definition
Allyship	Individuals engaged in ongoing, self-reflection examining how their choices impact Indigenous peoples both directly and indirectly. Allies promote the rights of non-dominant groups and work to eliminate social inequality that allies benefit from. Allies offer support through the establishment of meaningful relationships with people and communities of the non-dominant group that they are working with. It is about supporting and not leading the work for social change and inequitable systems and institutions and this is in keeping with the ‘Nothing for Us without Us’ principle.
Decolonization	The process of critically examining polices and deconstructing institutions that perpetuate the privilege and superiority of the dominant culture, while at the same time, also valuing and revitalizing Indigenous knowledge and Indigenous ways of being [28].
Indigenization	The “process of naturalizing Indigenous knowledge systems and making them evident to transform spaces, places, and hearts. … Indigenous knowledge systems are embedded in relationship to specific lands, culture, and community …. Because they are diverse and complex, Indigenization will be a unique process for every … institution. It is important to note that Indigenization does not mean changing something Western into something Indigenous. The goal is not to replace Western knowledge with Indigenous knowledge, and the goal is not to merge the two into one. Rather, Indigenization can be understood as weaving or braiding together two distinct knowledge systems … to understand and appreciate both. … it refers to a deliberate coming together of these two ways of knowing” [28].
Reconciliation	The process of “addressing past wrongs done to Indigenous peoples, making amends, and improving relationships between Indigenous and non-Indigenous people to create a better future for all. … With reconciliation between Indigenous and non-Indigenous people, … we are also talking about a relationship between multiple groups of people and between many generations over hundreds of years. Clearly, the onus for this action is on the party that perpetrated the harm, which in this case is settler society. For Indigenous peoples it means revisiting experiences of trauma and becoming open to forgiveness, and for settlers it involves gaining in-depth understanding of one’s own relation to Indigenous peoples and the impacts of colonization, including recognizing settler privilege and challenging the dominance of Western views and approaches” [28].

#### Nothing for us without us – facilitating an indigenous review process

The HEIA Indigenous Lens Tool [29] was developed through a collaborative and iterative process with stakeholders at CCO and with representatives from Indigenous community-based organizations. The Indigenous Cancer Care Unit (ICCU) at CCO served as the reference organization during the development of the tool. The ICCU works closely with Regional Indigenous Cancer Leads and Indigenous Cancer Navigators in all regions of Ontario who are experts in the provision of cancer care to Indigenous persons in the province. Additionally, the Joint Ontario Indigenous Cancer Committee (JOICC) provides guidance and advice to CCO with the aim of decreasing the incidence of cancer among Indigenous peoples living in Ontario. The committee is made up of Indigenous representatives from Ontario Political Territorial Organizations and provincial Indigenous organizations, Indigenous Elders and Knowledge Keepers who provide spiritual guidance and support, and members from CCO and Canadian Cancer Society [30, 31]. These representatives and organizations (N = 55 individuals) contributed to the development of the tool through virtual document sharing and a series of web-based or in person meetings. It is anticipated that the integration of the HEIA Indigenous Lens Tool into CCO operations will be facilitated by these stakeholders, who are embedded into CCO operations through the ICCU.

#### Application of the HEIA Indigenous Lens Tool

This Indigenous Lens Tool is positioned as a supplement to the HEIA Tool and as such provides a framework to think about and understand health equity for Indigenous peoples living in Canada. The document is in no way meant to be comprehensive or representative of the diverse health care experiences of Indigenous peoples living in Canada. Nor does it provide a comprehensive representation of the social determinants that surround health and well-being of Indigenous peoples living in Canada. Indeed, the concepts of equity presented here and in the HEIA Tool, as well as foundational concepts to equity such as the social determinants of health, need be to be recognized as Western or colonial constructs and not reflective of concepts of well-being and inter-relatedness of individuals, families, communities, and the environment that are common among many Indigenous knowledge systems. Rather, this document provides a first step to support development of policies and programs that recognize and uphold the rights of Indigenous peoples living in Canada to health and well-being.

Four scenarios, with supporting documentation, were developed to bring an Indigenous lens to the principles outlined in the HEIA Tool (Supplement and Table 3). These scenarios are in no way meant to replace meaningful engagement with Indigenous peoples and they are in no way meant to be comprehensive or representative of complex and varied experiences of diverse Indigenous peoples. Rather, the scenarios intend to provide context for each step of the HEIA Tool using fictitious examples and thereby facilitate application of the equity framework to programs and policies in the organizations using the tools. In the Indigenous Lens Tool, each scenario is followed by a discussion of the steps in the HEIA Tool that were highlighted in that scenario as well as tips and resources that may be useful.

**Table 3:** Summary of Indigenous Lens Tool scenarios

HEIA Tool Steps	Scenario
1a – Populations 2 – Potential Impacts	You are the Executive Director of an urban cancer centre and you are engaged in an update of existing policies with a focus on care of diverse patient populations. Planning proceeds and, after extensive consultations, the final draft is ready to be tabled. Your assistant says she received a patient feedback form that you need to see before presenting the final draft of the revised policies to the board. After the demographic data section, which identified the patient as Indigenous, the form reads: “I’ve been seeing my doctor for four years. I don’t like to come to the city because it’s so big, so sometimes I avoid going to see my doctor. Everyone is so busy in the city, and it can be hard to ask questions. My aunty said maybe they have a cancer navigator in the city who could help me. My aunty got the nursing station to arrange for an escort to come with me to the city, to help me out. Having someone come with me helps, but I still don’t like to stay long, because I want to get home to be with my family.” You look at your assistant in shock. Your research and consultations for the policy review had never identified Indigenous peoples as a population that was served by your cancer centre.
1b – Determinants of Health 2 – Potential Impacts 3 – Mitigation	You are a member of a provincial cancer screening policy team assessing the effectiveness of a new component of a cancer screening program – mailed letters that invite screen-eligible people to participate in colorectal cancer screening based on the address associated with their provincial health insurance card. Health administration databases were used to determine which proportion of letters sent out are returned to your organization (e.g., because the address information is incorrect) and whether the letters have had an impact on colorectal cancer screening participation rates. You find that less than 10% of mailed letters are returned, and there is a modest increase in screening participation after the introduction of the letters. You are pleased to report that the correspondence initiative may be positively impacting cancer screening participation and present the findings of your assessment at an internal meeting. A colleague from the Indigenous Health Team at your organization asks how this initiative is working for Indigenous peoples, as they are aware that their incidence of colorectal cancer is significantly higher. Through regional engagement with Indigenous communities, they have heard that some community members are not familiar with the screening letters at all, while others report not knowing what to do about the letters when they get them. You are surprised by this comment and realize that your assessment of the correspondence program did not identify this as an issue. An assessment of how the correspondence initiative impacts specific populations who are ‘under or never screened’ was not completed. You lose confidence that the new correspondence initiative is capturing all the screen-eligible people in the province.
2 – Potential Impacts 4 – Monitoring	The cancer centre leadership identified equity as a guiding principle in a planned overhaul of programming and has employed the HEIA Tool to guide program development. Equity, with respect to Indigenous patients was a particular focus as this group was identified as having lower rates of screening participation and lower rates of follow up after a positive screen. Furthermore, Indigenous patients were found to under-utilize programs and services at the cancer centre compared to the general population. The Indigenous Advisory Committee at your hospital was involved from the outset and recommended substantive changes to existing policies and programs that were implemented. A suite of patient-based key performance indicators was developed to monitor the impact of these changes. You are a program manager at the regional cancer centre attending the first annual screening performance meeting following implementation of the new initiatives. For the first time, screening and cancer centre utilization data are available for Indigenous communities served by your centre. The leadership team is pleased to hear that participation in programs located at the cancer centre has increased and that patient evaluations have also been positive. The data for screening, however, is less favourable. There has been no change in screening rates among Indigenous patients and no improvement in the rates of follow up after a positive screen. There is discussion around the table as to why the programmatic changes and the focus on equity did not yield results. You are tasked with understanding what happened.
2 – Potential Impacts 5 – Dissemination	You work at a regional cancer centre that has developed a wellness and cancer survivorship program that offers supports to patients recovering from cancer and their families. You used the HEIA Tool at the outset of program development, to ensure that your program will serve your regional cancer centre community equitably. You identified Indigenous peoples as a key population that might experience unintended impacts of the program (HEIA step 1a). Through your own research, discussion with Regional Cancer Program (RCP) team members with expertise in Indigenous health, and through direct engagement with the diverse urban and rural Indigenous communities in your region, you have pulled together resources and worked with Indigenous partners to co-develop programming that reflects the diversity of languages, cultural practices, and systems of knowledge among the RCP population (HEIA Steps 1b, 2 and 3). You have also worked with community partners to set up an ongoing data collection and monitoring process so that you will receive regular data about program participation and experiences, in an effort to support the evolution of the program as necessary to continue to meet the needs of the diverse population.

## Conclusions

The purpose of creating an Indigenous Lens Tool is to provide additional guidance when thinking about equity, health and Indigenous peoples. It is intended as a support for those using the HEIA Tool in further engaging with diverse Indigenous health experiences and perspectives, so they can more effectively identify and consider unintended impacts of initiatives on health and wellbeing of Indigenous peoples. In particular, this supplement to the HEIA Tool was developed to provide an Indigenous lens to view equity as it relates to policy and program development at CCO – an organization that identified health equity as an important strategic objective in the Ontario Cancer Plan V [30]. That being said, the principles described in this document are also relevant to Ontario Health, which has developed its own Equity, Inclusion, Diversity and Anti-Racism Framework [32], and other health and social service applications. However, to truly understand equity from diverse Indigenous perspectives, more rigorous and comprehensive Nation-specific analyses and tools that are initiated, developed and disseminated by Indigenous peoples, communities and Nations themselves are required. The Indigenous Lens Tool supplement to the HEIA Tool serves as a placeholder until more culturally relevant equity resources are available.

## Data Availability

The datasets used and/or analysed during the current study are available from the corresponding author on reasonable request.

## Abbreviations

CCO: Cancer Care Ontario
FNIM: First Nations, Inuit and Métis
HEIA: Health Equity Impact Assessment
ICCU: Indigenous Cancer Care Unit
JOICC: Joint Indigenous Cancer Committee
TRC: Truth and Reconciliation Commission
